# Adaptive multichannel FES neuroprosthesis with learning control and automatic gait assessment

**DOI:** 10.1186/s12984-020-0640-7

**Published:** 2020-02-28

**Authors:** Philipp Müller, Antonio J. del Ama, Juan C. Moreno, Thomas Schauer

**Affiliations:** 1 Technische Universität Berlin, Berlin, Germany; 2 Universidad Rey Juan Carlos, Madrid, Spain; 3 Instituto Cajal, Spanish National Research Council (CSIC), Madrid, Spain

**Keywords:** Multichannel neuroprosthesis, unctional electrical stimulation, Iterative learning control, Automatic gait assessment, Real-time motion analysis, Joint angle tracking, Gait support

## Abstract

**Background:**

FES (Functional Electrical Stimulation) neuroprostheses have long been a permanent feature in the rehabilitation and gait support of people who had a stroke or have a Spinal Cord Injury (SCI). Over time the well-known foot switch triggered drop foot neuroprosthesis, was extended to a multichannel full-leg support neuroprosthesis enabling improved support and rehabilitation. However, these neuroprostheses had to be manually tuned and could not adapt to the persons’ individual needs. In recent research, a learning controller was added to the drop foot neuroprosthesis, so that the full stimulation pattern during the swing phase could be adapted by measuring the joint angles of previous steps.

**Methods:**

The aim of this research is to begin developing a learning full-leg supporting neuroprosthesis, which controls the antagonistic muscle pairs for knee flexion and extension, as well as for ankle joint dorsi- and plantarflexion during all gait phases. A method was established that allows a continuous assessment of knee and foot joint angles with every step. This method can warp the physiological joint angles of healthy subjects to match the individual pathological gait of the subject and thus allows a direct comparison of the two. A new kind of Iterative Learning Controller (ILC) is proposed which works independent of the step duration of the individual and uses physiological joint angle reference bands.

**Results:**

In a first test with four people with an incomplete SCI, the results showed that the proposed neuroprosthesis was able to generate individually fitted stimulation patterns for three of the participants. The other participant was more severely affected and had to be excluded due to the resulting false triggering of the gait phase detection. For two of the three remaining participants, a slight improvement in the average foot angles could be observed, for one participant slight improvements in the averaged knee angles. These improvements where in the range of 4^*circ*^at the times of peak dorsiflexion, peak plantarflexion, or peak knee flexion.

**Conclusions:**

Direct adaptation to the current gait of the participants could be achieved with the proposed method. The preliminary first test with people with a SCI showed that the neuroprosthesis can generate individual stimulation patterns. The sensitivity to the knee angle reset, timing problems in participants with significant gait fluctuations, and the automatic ILC gain tuning are remaining issues that need be addressed. Subsequently, future studies should compare the improved, long-term rehabilitation effects of the here presented neuroprosthesis, with conventional multichannel FES neuroprostheses.

## Background

People who had a stroke or have a Spinal Cord Injury (SCI) experience impaired motor control. Limited locomotion function can have a big impact on the health and overall life quality of these persons. After a stroke or accident, the persons go through a rehabilitation period in which they try to regain as much of their former motor function as possible. After rehabilitation, stroke survivors or people with SCI might continue to see a physiotherapist. Over the last several decades Functional Electrical Stimulation (FES) has proven to be a useful tool in rehabilitation [1–3]. FES can help with basic muscle training [4], can initiate or amplify motion, and can provide sensory feedback [5]. Compared with a passive orthosis, FES does not limit the range of motion or the use of muscles [6]. While exoskeletons can offer higher forces and support, FES is comparatively light weight, less expensive and more physically engaging. The main disadvantages of FES are the limited amount of achievable force (especially using non-invasive surface electrodes) [7, 8], the complexity of motion control using stimulation, the increasing discomfort with higher stimulation intensities and the rapid muscular fatigue of the artificially activated paretic muscles [9]. FES-based neuroprostheses are therefore best suited to lightly affected persons or in combination with robotics.

The first FES-based neuroprosthesis was a drop foot stimulator introduced by Liberson et al. in 1961 [10], in which the stimulation of the tibialis anterior muscle was triggered on and off by a foot switch attached to the person’s heel. Commercial drop foot systems available today still follow the same basic principle: heel-rise and initial contact of the foot are detected using either a foot switch or an inertial sensor, and the stimulation profile is a square or trapezoidal pattern during the swing phase (e.g., the Odstock Dropped-Foot Stimulator produced by Odstock Medical Ltd in the UK).

The single channel tibialis anterior stimulation was later extended to multichannel neuroprostheses, in which more muscles of the gait muscle complex were included in the stimulation [11–13]. In these studies, gastrocnemius, hamstrings, quadriceps, gluteus maximus, gluteus medius and even shoulder muscles were stimulated. Kim et al. were able to show that multichannel stimulation (of gluteus medius and tibialis anterior) is superior to single channel stimulation (of only the tibialis anterior) in terms of gait improvement [13].

A main issue with this first-generation FES prosthesis is that the stimulation patterns and timings are rigid and can only crudely and manually be adjusted to the needs of the individual person. Recent research has tried to address this issue. One approach is to use more true to life stimulation patterns, replacing the trapezoid or rectangular patterns. O’Keeffe et al. and Breen et al. derived a stimulation pattern for the tibialis anterior muscle from Electromyography (EMG) data of healthy subjects [14, 15]. The fixed stimulation pattern was triggered with a foot switch and resampled to the current estimated step duration. Meng et al. extended this approach to include four muscles, the quadriceps, hamstrings, tibialis anterior and gastrocnemius muscles [16]. In a previous study [17], the EMG muscle activity of ten healthy subjects during gait was recorded in relation to five gait events. This recorded EMG activity was converted to a stimulation intensity pattern and played back at the corresponding gait events of each individual participant. The method was tested on seven healthy subjects.

The remaining issues, however, are that the shapes of the stimulation patterns are not adjusted to the individual person’s needs, and that the intensities have to be manually tuned for each subject. Chia et al. and Ferrante et al. went one step further by deriving stimulation patterns from the gait EMG activity of the individual subject [18, 19]. This was done in a separate session in which the EMG activity was measured in relation to six gait events. By comparing the measured EMG data to data obtained from healthy subjects, stimulation patterns could be derived. In a preliminary evaluation with two stroke survivors, a gait improvement could be shown after four weeks of training with the stimulation. An advantage of this approach is that after the extensive calibration session, no additional sensors, besides the foot switch or inertial gait phase detection, are necessary. The stimulation patterns, however, are calibrated to the circumstances at the time of the measurement, and cannot adapt to changes in gait caused by factors such as fatigue, mental focus and longer term rehabilitation improvement.

As well as orthoses, exoskeletons, robotics and FES, EMG biofeedback is a method to improve rehabilitation therapy. In EMG biofeedback the EMG activity of one or multiple muscles is measured and directly fed back to the subject in the form of audio or video signals. This enables a direct feedback of the subjects’s performance. Moreland et al. showed in a review of eight studies, that EMG biofeedback performs better compared to conventional therapy of the lower extremities [20]. Lourenção et al. were able to show that combined FES and EMG biofeedback based rehabilitation performed better than an exclusive FES therapy for the upper extremities [21]. Cozean et al. showed that applying EMG biofeedback during gait, together with FES, performed better than exclusive FES or conventional therapy [22]. Laufer et al. analyzed the potential of sensory electrical stimulation in which the stimulation is felt but no muscle recruitment is produced [23]. Laufer et al. concluded, that the combination of sensory electrical stimulation and active training has the biggest potential for improved rehabilitation. However, due to limited studies the long-term results were inconclusive. The presented studies on EMG biofeedback suggest that direct feedback of the persons’s performance is beneficial to therapy and that FES is a valid choice for biofeedback. The aforementioned neuroprostheses, which use unchanging (aside from resampling) gait event triggered stimulation patterns, do not adapt to the subject’s performance and, therefore, miss out on the additional therapeutic benefits of biofeedback.

A different FES neuroprosthesis approach is to adapt the stimulation patterns in real-time to the gait of the subject. This, however, needs a form of measurement of the current gait of the subject, meaning that additional sensors are necessary. Classical feedback control (e.g., PID control) is not suitable in gait applications due to the slow dynamics between stimulation onset and motion. Chen et al. specified a muscle independent latency of approximately 0.1 s between stimulation and the generated force in the muscle [24]; Müller et al. and Seel et al. identified a delay of 0.2 s between stimulation and joint angle response [25–27]. For example, for a healthy person walking at 3 km/h, the duration of the swing phase would be approximately 0.25 s [28] (assuming a 40 % swing). Thus, a direct feedback control of the joint angle during gait cannot be achieved by FES.

Fortunately, gait is a repetitive motion, and therefore deficits of the last step can be accounted for in the next step. Using information from the previous cycles to influence the current cycle is generally referred to as learning control. Relevant methods of learning control include Iterative Learning Control (ILC), used for full trajectory control, and Run To Run Control (R2R), used for single parameter control [29].

Franken et al. used R2R (in this case is was called cycle-to-cycle control) to automatically tune the single parameter of the stimulation duration of the hip flexor muscle at every step, by measuring the hip angle range [30]. ILC was first used together with FES by Dou et al. to control the elbow flexion/extension angle [31]. Instead of a single parameter, the full stimulation pulse width trajectory was controlled, enabling full control of the elbow flexion. Nahrstaedt et al. were the first to apply ILC during gait on the tibialis anterior muscle [32]. Hughes et al., Freeman et al. and Meadmore et al. further investigated into ILC strategies for the upper limbs [33–35]. Seel et al. used ILC to control the tibialis anterior and fibularis longus muscle, achieving physiological dorsiflexion and eversion of the foot in walking stroke survivors [26, 27]. This was achieved by identifying the coupling between, on the one hand, the two muscles and, on the other, the dorsiflexion and eversion angles. With this knowledge, two separate ILCs could be used for each joint angle.

**Fig. 1:**
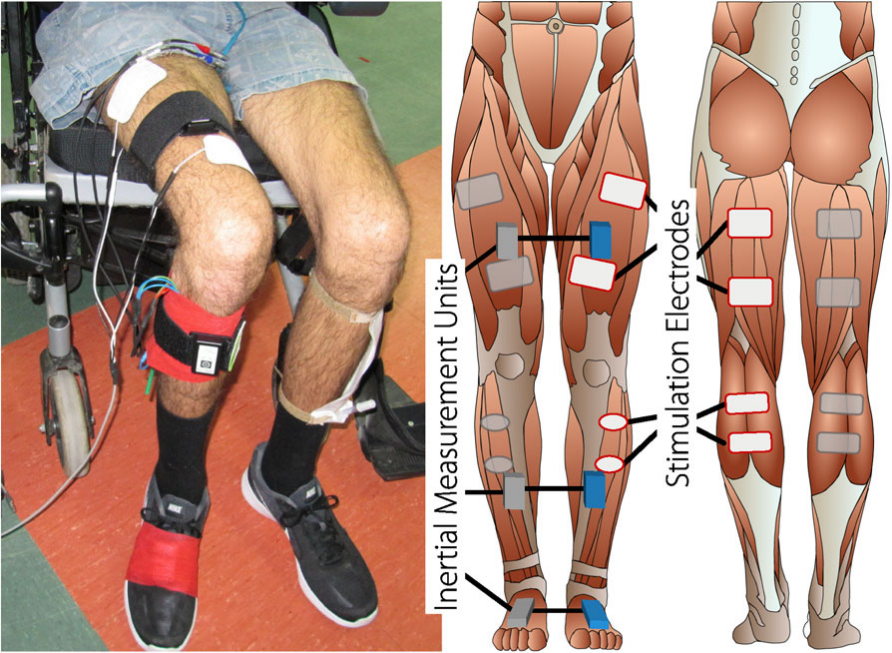
Placement of the neuroprosthesis (single leg setup) on one of the participants (left image) and the schematic placement of stimulation electrodes and sensors (right image). The neuroprosthesis supports the stimulation of four muscle groups (quadriceps, hamstrings, tibialis anterior and gastrocnemius) to control the knee and foot motion during gait. The control of each leg is independent, and therefore the double leg setup is a duplication of the single leg setup. Due to the limitations of the wireless sensors, the single leg setup was used in this work

For gait applications, so far only ILC control of dorsiflexion muscle groups during the swing phase were achieved. In a preliminary work, we studied the system dynamics of the knee flexion/extension angle when stimulating during different phases of the gait. We established a first version of an antagonistic knee ILC, which was tested on eight healthy subjects [25]. We are now developing a learning FES neuroprosthesis that supports the four antagonistic muscle groups of the upper and lower leg by assessing the knee and foot angle (the basic setup can be seen in Fig. 1). In this paper we present the development of an automatic stepwise joint angle assessment, the development of a walking speed independent iterative learning controller, the implementation of the neuroprosthesis, a first test with four people with SCI and the evaluation of the test.

## Automatic gait assessment

The purpose of the automatic gait assessment is to provide the learning control with continuous error signals for the knee and foot joint. These error signals should encode at which points in time the subject needs more flexion or extension. This is comparable to a therapist looking at the person’s gait and noting in which phase of the gait cycle deficits occur and of what intensity they are.

A way to systematically measure those deficits is to measure the joint angles, gait cycle by gait cycle, and to compare them with a desired reference. Several methods for measuring joint angles and gait phases using Inertial Measurement Unit (IMU) data can be found in literature. To automatically find references that match the different gaits and step cycle durations of people with pathological gait proved to be challenging. New methods of automated reference generation are proposed in this publication.

### Measuring joint angles and gait phases

There are multiple IMU-based real-time gait phase detection algorithms available in literature [36–38]. In this paper the foot mounted inertial sensor gait phase detection from Müller et al. was used [39]. This algorithm can detect four gait events per foot sensor: initial contact, foot-flat, heel-off and toe-off.

Inferring joint angles from raw IMU data is a well known procedure, see for example, [40–42]. When compared with optical reference systems, for IMU based joint angle measurements in the sagittal plane, the precision was found to be in the range of 3^*circ*^or lower [40–42]. One problem is that three dimensional orientations can only directly be obtained by using magnetometer measurements. Those measurements, however, are often heavily disturbed. By using mechanical constraints of body and gait it is possible to omit the use of magnetometers with certain tradeoffs. Different methods of varying efficacy are available in literature. Here we will focus on a plain and robust solution, which has adapted on some of these previous methods. Due to the plentitude of available publications, the chosen method will be described very briefly in this paper.

The sensors are assumed to be aligned (x-axis along the limb and z-axis facing along the knee joint axis or the ankle dorsi/plantarflexion joint axis). Errors in the alignment can lead to errors in the joint angle measurement, yet, Fennema et al. found that IMU alignment was acceptably repeatable for the knee joint [43]. Depending on only one sensor, the foot-to-ground angle is expected be less sensitive to alignment errors.

For the knee angle, the angle between the gravity vector of the upper leg sensor and the gravity vector of the lower leg sensor, projected to the sagittal plane, is obtained. The part of the measured angular velocities of the upper and lower leg that points along the knee joint axis is subtracted to form the relative knee joint angular velocity. This value is then integrated and fused together with the estimated angle, based on the accelerations using a variable weight. The value of the weight is determined by how close the norm of the accelerations resemble gravity for the last five samples. This rating ensures that acceleration peaks generated by the gait do not influence the angle estimation.

**Fig. 2:**
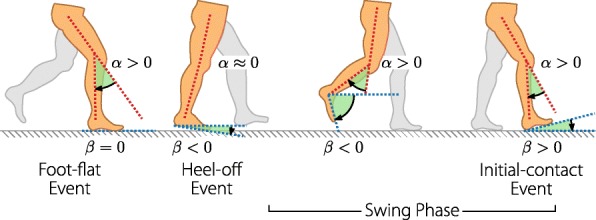
Joint angle definitions of the knee angle *α* and the foot-to-ground angle *β*. The knee angle is defined zero for a straight leg and positive for knee flexion. The foot-to-ground angle is zero when the foot is parallel to the ground and positive when the forefoot is pointing upwards

The foot-to-ground angle was obtained by integrating the part of the measured foot angular velocity that points along the ankle dorsi/plantarflexion joint axis. This value is set to zero with each foot-flat event. To correct the drift of the angular velocity measurement, the foot-to-ground angle vector between two foot-flat events was retroactively changed so that the first and last joint angle value equals zero. This could be achieved by subtracting a sloped line from the trajectory. Figure 2 illustrates the definition of the knee and foot-to-ground angle. The calculated foot-to-ground angle is only correct when the pitch of the foot during the foot flat phase is close to the pitch of the previous foot flat phase; ergo, this method would not be suitable when walking on uneven terrain.

### Physiological joint angle reference bands

**Fig. 3:**
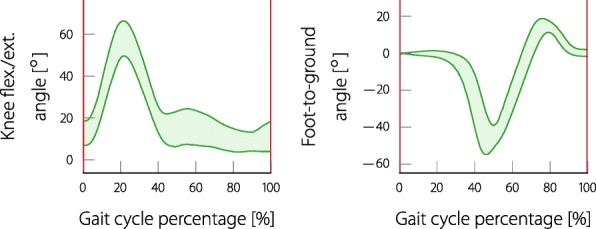
The knee and foot-to-ground angle reference bands. The bands are the standard deviations of the mean joint angles obtained from measurements with healthy subjects. The knee reference starts and ends at the heel-off event whereas the foot reference starts and ends at the foot-flat event

In order to be able to evaluate pathological gait, a reference must first be defined. We chose to measure the gait of healthy persons and used this data as a reference for a good gait. Four people (aged 38.5 ±5.5 years) were asked to walk with four different speeds (1.5, 2, 2.5 and 3 km/h) on a treadmill. The measured joint angles were cut into gait cycles using the events of the gait phase detection. Hence, for both, the knee and foot angle, there are four different options to define the start of the gait cycle. Since we want to compare the angle of one gait cycle to a reference, it would be beneficial if the start and end of the angle trajectory were at a predictable value. For the foot-to-ground angle, the angle is zero by definition at the foot-flat event (see Fig. 2). For the knee angle, there is no phase where the angle is previously known. The heel-off event was chosen as a reliable event in which the knee is relatively straight for most subjects, as hinted in Fig. 2. Each measured joint angle of one gait cycle was resampled to a duration of 100 samples. Using the data of all subjects, the mean and standard deviation for a "healthy" foot and knee angle were determined as presented in Fig. 3.

When using the obtained reference bands to assess the gait of a person, the setting should be similar to the setting of when the reference bands were recorded. In our case this would be the walking on level ground with moderate walking speeds. Ascending stairs, shuffling, running or walking on uneven terrain requires different motion sequences and, accordingly, different sets of joint angle references.

The standard way in control engineering is to directly compare (subtract) the measured trajectory with the reference trajectory and act on the resulting error. For the step assessment, two issues arise: firstly, because the subject freely chooses the walking speed, the step duration will vary; secondly, a healthy gait varies and does not exactly follow a fixed trajectory, so there should be no errors resulting from natural variations. The intuitive solution to the first issue is to squeeze/stretch (resample) the reference trajectory to the duration of the current step. For the second issue instead of a reference trajectory, a reference band can be used which is defined by the mean and standard deviations of the above measurements. The error of the joint angle in relation to the reference band is defined as zero when in the band, otherwise it is defined as the distance to the band. This means that if the joint angle stays within a physiological range, the error remains zero. For a joint angle trajectory $\phantom {\dot {i}\!}\mathbf {y} = [y_{1} \ldots y_{N_{\text {step}}}]^{T}$, and the upper reference trajectory $\phantom {\dot {i}\!}\mathbf {r}_{\text {upper}} = [r_{\text {upper},1} \ldots r_{\text {upper}, N_{\text {step}}}]^{T}$, and the lower reference trajectory $\phantom {\dot {i}\!}\mathbf {r}_{\text {lower}} = [r_{\text {lower},1} \ldots r_{\text {lower}, N_{\text {step}}}]^{T}$, the elements of the error trajectory $\phantom {\dot {i}\!}\mathbf {e} = [e_{1} \ldots e_{N_{\text {step}}}]^{T}$ are defined as:

1 $$\begin{array}{*{20}l} e_{i} &= \left\lbrace \begin{array}{ll} r_{\text{upper},i} - y_{i} & \ \text{if}\ y_{i} > r_{\text{upper},i}\\ r_{\text{lower},i} - y_{i} & \ \text{if}\ y_{i} < r_{\text{lower},i}\\ 0 & \ \text{otherwise} \end{array}\right.\\ &\qquad\forall i \in [1 \ldots N_{\text{step}}],  \end{array} $$

where *N*_step_ is the number of samples of the measured step.

**Fig. 4:**
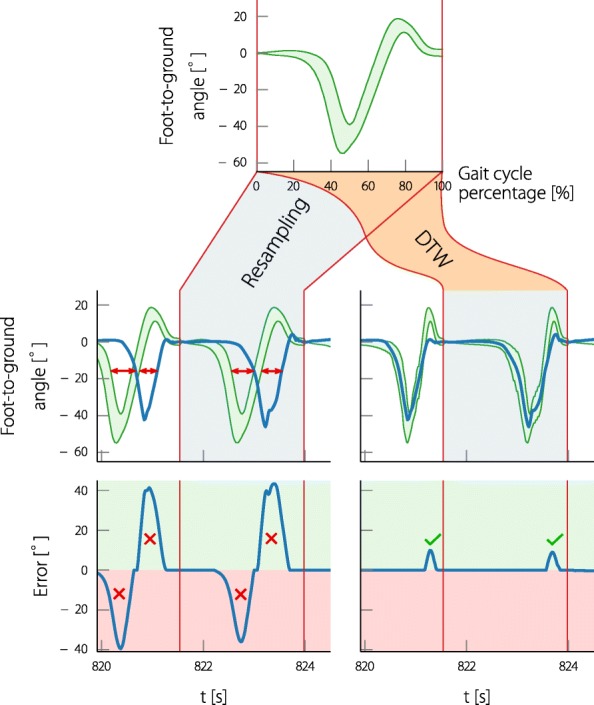
Two methods are proposed to fit the reference bands to the step of the subject. On the left: the resampling of the reference to the current duration of the step. And on the right: using dynamic time warping to adjust the reference to the joint angle of the current step. The second row shows the measured foot-to-ground angle of a subject () and the fitted reference bands (). The third row shows the resulting error with respect to the reference bands (). The problematic shift of the joint angle to the reference band obtained by the first method and the resulting error, is indicated (). The missing dorsiflexion of the subject during the swing-phase is consistent with the error obtained from the warped reference ()

The effects of applying the resampling to the reference band of the foot-to-ground angle of a pathological subject’s gait cycle can be seen in Fig. 4. The introduced physiological range reference band (upper row) is resampled to the duration of the measured step of the subject (center left) and the resulting error is shown (lower left). When looking at the joint angle and the reference it becomes evident that the motion of the subject follows almost the same motion as the reference, but is somehow delayed. The range of motion however is almost identical, only in the positive plane the motion of the subject is of smaller range, indicating missing dorsiflexion. Due to the time shift of the reference to the subject’s angle, the resulting errors are enormous, suggesting an extreme amount of push off and dorsiflexion missing in the subject’s gait. The errors do in no way resemble the subject’s lack of motion but only the subject’s lag or temporal distortion of the gait. The aim of the neuroprosthesis is to support the persons in their individual motions and not to force them to a completely new pattern. In the previous works, for example, [26, 27], the motion was only rated for the swing phase and the stimulation was only pulling the angle in one direction. The reference was also tuned separately for each subject. For a general solution, which works for antagonistic muscle stimulation in all gait phases, a method that automatically adjusts the reference to the gait of the individual subject has to be found.

### Adjusting the reference to the subject’s gait using dynamic time warping

With the previously presented resampling method, the reference is sometimes ahead and sometimes lagging in relation to the measured joint angle, indicating that there is a problem with the timing of the reference signal. A well-known method (from signal processing, especially speech recognition) that addresses the comparison between two signals that are warped in time, is Dynamic Time Warping (DTW) [44, 45]. By accelerating or decelerating the signal time, DTW finds the optimal time sequence, so that the two signals become the most similar. This means that a signal can be stretched and squeezed in the time domain so that it optimally fits to another signal, while still providing the same sequence of values.

**Fig. 5:**
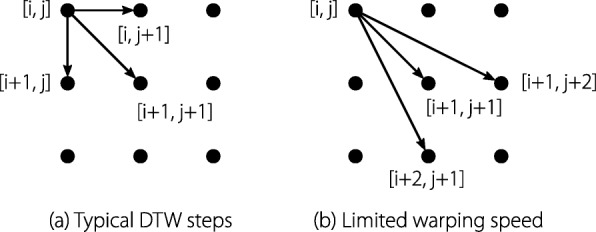
Fundamental steps of the dynamic time warping algorithm. The most common steps (**a**) allow infinite stretching, whereas the steps shown in (**b**) limit the maximum and minimum warping speed to 1.5 and 0.5, respectively

In essence, DTW determines the optimal path in a matrix in which each element represents the error between the i’th element of signal 1 and the j’th element of signal 2. The path through the matrix is a composition of elementary steps and DTW finds the sequence of steps which yield the lowest cost. The elementary steps used in the standard form of DTW are shown in Fig. 5a. Using these elementary steps allows infinite acceleration and deceleration of a signal (by going vertical/horizontal) which can lead to extreme and unnatural results. It is possible to constrain the solution of DTW: firstly, by limiting the space where DTW can act inside the matrix (by using Sakoe-Chiba bands and Itakura parallelograms [44, 45]); secondly, by enforcing a minimum and maximum speed of time by changing the elementary steps. The first solution cannot limit the maximum and minimum warping speed, whereas the second solution is limited by a discrete selection of steps. The elementary steps which are typically used with DTW are presented in Fig. 5a. The steps shown in Fig. 5b limit the warping speed to a minimum of 0.5 and a maximum of 1.5.

**Fig. 6:**
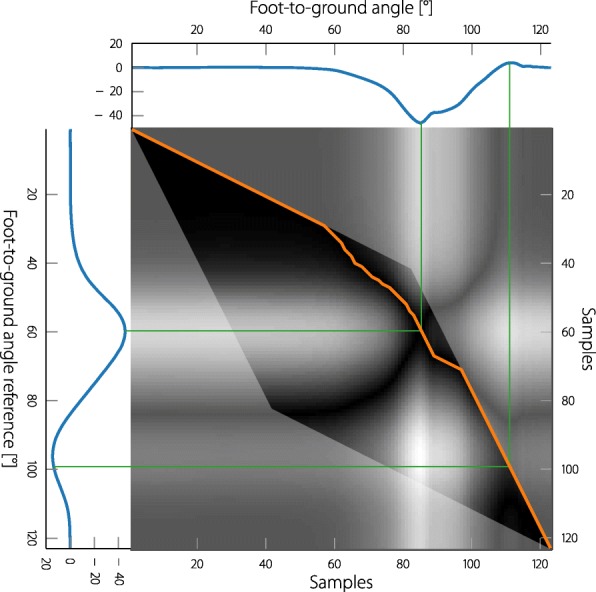
The distance matrix between the foot-to-ground angle of one step of a SCI participant and the mean foot reference angle. Each sample of the reference is compared to each sample of the measurement and the distance of the two signals visualized as a pixel of the matrix. Black indicates no distance and white indicates the largest distance. The area that can be reached by the DTW is limited by the chosen fundamental steps. For the steps chosen, the grayed-out area cannot be reached by the DTW. () is the resulting optimal warping path and () are two examples of matched samples

The matrix showing the absolute errors of two signals is presented in Fig. 6. The participant’s foot-to-ground angle introduced in Fig. 4 is compared to the resampled mean of the foot-to-ground angle reference presented in Fig. 3. The elementary steps from Fig. 5b are used, this automatically excludes the grayed out area, which can only be reached by a faster warping speed. The resulting optimal warping path first compresses the reference signal with the lowest speed possible until the push-off, then progresses along the valley until terminal swing, where it stretches the reference with the highest possible warping speed. If the person, for example, has no distinct dorsiflexion during the terminal swing phase, the DTW would not find a similarity to the foot-to-ground angle of the reference signal during that phase and would try to skip as quickly as possible through this section of the reference. By limiting the warping speed, the DTW cannot skip parts of the reference that are not showing in the joint angle. Even when the joint angle does not contain a positive foot-to-ground angle section, the warped reference will still contain a (shorter) version of its original section. This leads to a new reference that adapts to the subject’s gait, but at the same time enforces the motion of the healthy reference.

The DTW is always applied to the mean of the joint angle reference. The obtained warping information is then applied to the reference band as shown in Fig. 4 (right column). The resulting error now indicates missing dorsiflexion during swing phase, and the warped reference matches the motion of the participant.

The introduced DTW method can warp a signal in time; however, the start points of the two signals are defined to be concurrent, and the same applies to the end points. Hence, a delay of the joint angle to the reference at the start point or end point could not be corrected by the DTW. This problem can be avoided by defining the start and end points to positions in which the joint angles can be assumed to be within the reference band. As presented in the previous subsection, the heel-off event is selected as a trigger for the knee angle measurement, and the foot flat event as a trigger for the foot-to-ground angle measurement. With this configuration the foot-to-ground angle starts at zero per definition and the knee angle can be assumed to be close to zero.

### Resulting gait assessment

The resulting gait assessment procedure is as follows. The knee angle is cut into heel-off event based cycles and the foot-to-ground angle is cut to foot-flat event based cycles. A heel-off event based knee-angle reference band and a foot-flat event based foot-to-ground angle reference band was obtained (Fig. 3). These reference bands are defined by the standard deviations of the measurement of healthy subjects and are expected to resemble the ranges of healthy joint angles. For each knee angle cycle and each foot-to-ground angle cycle the corresponding reference is resampled to the duration of this cycle. The resampled reference is then matched to the respective joint angle using DTW. Fundamental DTW steps were chosen that limit the allowed warping speed (Fig. 5b). To obtain the new reference bands, DTW was applied to the mean reference angle. The upper and lower reference bands are then warped with the warping information (indexes) of the warped mean reference angle. The cycle error for knee and foot-to-ground angle can be acquired using the respective joint angles and reference bands as defined in (1).

## Basic principles of the neuroprosthesis

The aim of the adaptive neuroprosthesis is to assess each step of the subject and to adapt the stimulation pattern for the next step accordingly. The assessment is based on the knee flexion/extension angle and the foot-to-ground angle. The stimulation intensity patterns are continuous signals for all four muscle groups that are stimulated. The aim is to support the subject during the entirety of the gait, hence the stimulation patterns span over the entire step duration and the entire step is evaluated.

For people with remaining sensory function and low stimulation tolerance levels, the FES can only aid but never replace the voluntary muscle action. If the subject is stimulated at significantly different timings than his natural progression through the gait cycle, the stimulation does not support but disrupt the person. Hence, in our case, being in sync with the subject’s intentions is very important. Therefore, the healthy reference bands are warped to be synchronized with the subject’s gait. Subsequently, the inferred stimulation patterns will help the subject to reach the same range of motion as the reference joint angle bands, but cannot help to reach the same timings.

The gait phases are determined separately for each leg using the foot-mounted IMUs. This means that the neuroprosthesis for one leg is completely independent from the prosthesis of the other leg. Thus, by simply copying the soft- and hardware the neuroprosthesis can be extended from one leg support to double leg support. In this work, however, due to the wireless bandwidth limitations of the sensors, we support only one leg (the more affected leg).

As we learned in the previous section, the gait assessment is triggered with the heel-off event for the knee angle and the foot-flat event for the foot-to-ground angle. When the gait event arises the gait cycle error of the previous cycle can be determined for the respective joint angle. Our aim is to use ILC to determine a stimulation intensity pattern for the next cycle of the subject by utilizing the previous cycle error. Note that for both, the knee angle control as well as the foot-to-ground angle control, two separate stimulation intensity patterns have to be established due to the antagonistic muscle pairs.

**Fig. 7:**
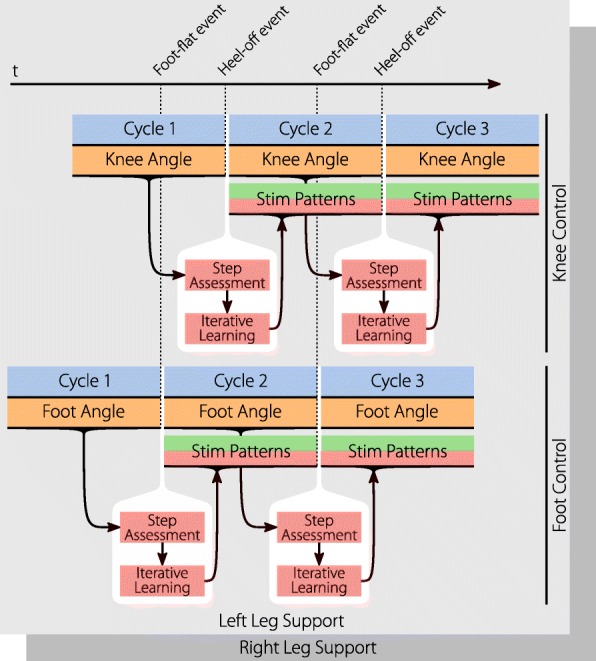
The basic workings of the neuroprosthesis: The knee angle is recorded and, with the heel-off event, passed to the gait assessment. The ILC learns new stimulation patterns from the resulting error of the assessment. These stimulation patterns are instantly applied until the next heel-off trigger (or the end of the pattern). The foot-to-ground angle control is working equivalently, but is triggered by the foot-flat event When supporting both legs, a counterpart is running at the same time for the other leg, using the gait events and joint angles of this leg

The cycle by cycle assessment, the learning, and the applying of stimulation are depicted in Fig. 7. Here the knee angle cycles, segmented by the heel-off event, and the foot angle cycles, segmented by the foot-flat event, can be seen. With each event, the step assessment and ILC is applied and a new stimulation pattern determined for the next cycle. Ideally the assessment and generation should happen in less than one sampling period, so that the new stimulation pattern can be immediately applied and the stimulation will not be interrupted. As stated before, when supporting two legs, a second copy is running in parallel, as implied by the second layer in the figure.

**Fig. 8:**
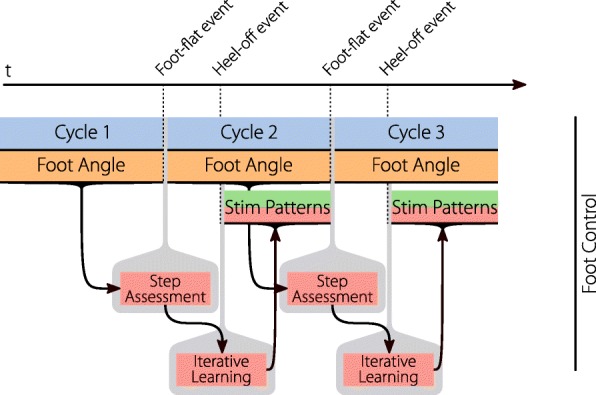
Second version of the foot control. Due to the duration from the foot-flat event to the stimulation during the swing phase, timing problems can arise in persons with irregular gait. This alternative triggers the stimulation at the heel-off event, which is closer to the swing phase, to ensure correct timing. This sacrifices the ability for push-off support since much of the support happens before the heel-off event

An issue with the triggered stimulation patterns is that step cycle duration variations can lead to timing errors with the stimulation intensity pattern. For example, if a step of the subject is much faster than previous steps, the stimulation in the middle of this step comes too late. Much of the stimulation happens during the swing phase. The foot-flat event is relatively far from the beginning of the swing phase compared with the heel-off event. For persons showing big gait variations the current version of the foot-to-ground angle control can lead to timing errors. For this group, a second version of the foot control was introduced and is shown in Fig. 8. The gait assessment is triggered, as usual, with the foot-flat event, but the ILC is triggered later at the heel-off event. The error trajectory from the gait assessment is shortened by the number of samples that passed from foot-flat to heel-off, and therefore the ILC creates a shorter stimulation pattern starting from heel-off. This solves the previous timing problems, at the cost of having no stimulation between the foot-flat and heel-off event. Hence, most of the subject’s push-off cannot be supported by stimulation in this case.

## ILC design

Two independent ILCs are used to control the antagonistic muscle pair of the knee and the foot of one leg. Each ILC is triggered with a gait event and provided with the error trajectory of the previous cycle from the gait assessment. The resulting control signals of each ILC are transformed into two stimulation intensity patterns for the two antagonistic muscles, using an input mapping strategy.

As in previous works [25–27, 32, 34], a P-type ILC is used (as thoroughly explained in [29]). In this work, however, two novel extensions are made: a new control strategy that is independent of the cycle duration, and an adaptation to reference bands.

### Input mapping

In order to use one Single Input Single Output (SISO) ILC controller per joint angle, each of the two antagonistic muscle pairs has to be mapped to one control signal. This control signal can be positive and negative, whereas the stimulation intensities of the muscles can be only positive. Dead zones can be avoided, and some joint stiffness gained by using cocontraction around the switching zone of one muscle to the other. A similar mapping was previously used in [25] and a detailed study of coactivation strategies can be found in [46]. The mapping is defined by

2 $$\begin{array}{*{20}l} q_{a,i} &= \left\lbrace \begin{array}{ll} q_{a0} + \frac{1}{k_{a}} u_{i} & \ \text{if}\ q_{a0} + \frac{1}{k_{a}} u_{i} > 0\\ 0 & \ \text{otherwise}  \end{array}\right. \\ q_{b,i} &= \left\lbrace \begin{array}{ll} q_{b0} + \frac{1}{k_{b}} u_{i} & \ \text{if}\ q_{b0} + \frac{1}{k_{b}} u_{i} < 0\\ 0 & \ \text{otherwise}, \end{array}\right.  \end{array} $$

where *u*_*i*_ is the control input at sample *i*, *q*_*a*,*i*_≥0 and *q*_*b*,*i*_≥0 are the corresponding stimulation intensities of the first and second muscle, *q*_*a*0_≥0 and *q*_*b*0_≥0 are the dead-zone stimulation intensities for a control input *u*_*i*_ of 0, and $\frac {1}{k_{a}} > 0$ and $\frac {1}{k_{b}} > 0$ are the stimulation gains in relation to the control input. This strategy allows cocontraction for low intensities, and fading to single stimulation for higher intensities. The input mapping can act as a static system inverse by setting *q*_*a*0_ and *q*_*b*0_ to the identified stimulation thresholds of the first and second muscle and *k*_*a*_ and *k*_*b*_ to the identified steady state gain of the corresponding muscle. Having a static system inverse as the input mapping means, that the ILC can be tuned to a system with an assumed gain of one and does not have to be customized for each subject (unlike the input mapping).

The validity of the static system inverse depends on the identified parameters. Different conditions (for example under load in contrast to swinging freely, or flexed in contrast to extended) can alter the properties of muscle groups. Müller et al. investigated the properties of the antagonistic knee muscles during different times of the gait cycle and compared them to a sitting pose [25]. Parameter identification experiments with 5 healthy subjects were conducted during walking and while sitting. Although noticeable variations of the identified parameters could be observed, it could be shown that the variations were still within the robustness margins of the applied ILC. Hence, parameters obtained from a sitting pose can be used to tune the ILC.

The stimulation intensity used in this publication is defined in the following way: since the intensity can be increased by increasing the stimulation pulse width or the stimulation current, the product of both, the charge, is chosen as intensity parameter. For a given charge *q* [µAs], the stimulation current *I* [mA] and the stimulation pulse width *p*_*w*_ [µs] are defined as:

3 $$\begin{array}{*{20}l} I &:= \sqrt{200 \, q}, \quad p_{w} := \sqrt{800 \, q}.  \end{array} $$

### Step duration independent control

The different forms of ILC control as described in [29] do not account for variable cycle duration. A straightforward modification is to choose a large enough ILC buffer and, during each cycle, to fill the error vector with zeros, so that it fits the buffer size. Seel et al. used this approach and were able to prove ILC stability (for a fixed reference) in this case [27]. This approach is a basic, if limited, way to deal with variable step durations. However, if a change from a small step duration to a bigger step duration occurs, this ILC-type will still apply the stimulation for short steps and has to learn the stimulation pattern of the now longer steps. Depending on the ILC tuning, this can take many iterations. This means that until the new stimulation pattern is learned, the stimulation timings will be out of sync with the subject’s gait, and the gait will not be supported and could be disrupted.

To address this problem, we designed an ILC that acts in the Gait Cycle Percentage (GCP) domain instead of the time domain. In the GCP domain, independent of the step duration, the step starts at 0 % and ends at 100 %. The error from the step assessment is transformed to the GCP domain, where the learning and storing of the ILC control signal also takes place. In order to apply the control signal, it has to be transformed back to the time domain using the current estimated step duration. Since we cannot foresee the duration of the next step, the estimation is based on the duration of the last step. Thus, the learning in the GCP domain will always be with the correct timings, since the previous step duration is known. However, the correctness of the scaling of the control signal is dependent on the step duration estimation.

The error from the previous cycle is acquired, as shown in the previous section, using the stepwise fitted reference bands. The first step is to limit the error, which ensures that unreasonable errors cannot have too much impact and also limits the rate of the learning:

4 $$\begin{array}{*{20}l} \bar{\mathbf{e}}_{k} = \overset{+e_{\text{max}}}{\underset{-e_{\text{max}}}{\text{sat}}} (\mathbf{e}_{k}),\quad \mathbf{e}_{k} = [e_{k,1} \ldots e_{k, N_{\text{step}, k}}]^{T}, \end{array} $$

where ±*e*_max_ defines the bounds of the error considered during the learning, **e**_*k*_ is the error vector from the previous cycle, *N*_step,*k*_ is the number of samples of the last cycle and $\bar {\mathbf {e}}_{k}$ the limited error.

The purpose of a Q-filter in ILC is to smooth out the control signal and thereby improve robustness. It was decided that the Q-filter should be applied in the time domain (as opposed to in the GCP domain). This ensures that short steps cannot produce steeper stimulation patterns compared with long ones. Applying the Q-filter and learning gain to obtain the new difference *Δ***u**_*k*_ to the control signal:

5 $$\begin{array}{*{20}l} \Delta \mathbf{u}_{k} = \lambda \mathbf{Q} \bar{\mathbf{e}}_{k}, \end{array} $$

where **Q** is the matrix of the Q-filter and *λ* the learning gain. This difference is now transformed to the GCP domain.

6 $$\begin{array}{*{20}l} \Delta \mathbf{u}_{k}^{*} = \underset{N_{\text{GCP}}}{\text{resamp}} (\Delta \mathbf{u}_{k}),\quad \Delta \mathbf{u}_{k}^{*} \in \mathbb R^{N_{\text{GCP}}}, \end{array} $$

where $\Delta \mathbf {u}_{k}^{*}$ is the control signal difference in the GCP domain, resamp is linear resampling and *N*_GCP_ is the number of samples in the GPC domain.

The learning of the new control signal now takes part in the GCP domain:

7 $$\begin{array}{*{20}l} \mathbf{u}_{k+1}^{*} = \overset{u_{\text{max}}}{\underset{u_{\text{min}}}{\text{sat}}} \left(\mathbf{u}_{k}^{*} + \Delta \mathbf{u}_{k}^{*} \right),  \end{array} $$

where $\mathbf {u}_{k+1}^{*}$ is the control signal for the upcoming cycle *k*+1. Since the stimulation intensities are limited to the preferences of each person, the control signal is limited in the same way (by choosing *u*_min_ and *u*_max_ correctly) to avoid ILC-windup.

To apply the control signal in the next cycle, it has to be transformed back into the time domain using the currently estimated step duration:

8 $$\begin{array}{*{20}l} \mathbf{u}_{k+1}^{\dag} &= \underset{\hat{N}_{\text{step}, k+1}}{\text{resamp}} (\mathbf{u}_{k+1}^{*}), \end{array} $$

where $\mathbf {u}_{k+1}^{\dag } = [u_{k+1,1}^{\dag } \ldots u_{k+1,\hat {N}_{\text {step},k+1}}^{\dag }]^{T}$ is the control signal and $\hat {N}_{\text {step},k+1}$ the estimated step duration.

One advantage of iterative learning control is that constant time delays can be easily compensated due to the prior knowledge of the error. In the classic ILC this is done by shifting the error vector **e**_*k*_ by *m* samples. In this case, after joining the error, the control signal vector is resampled to the GCP domain and subsequently resampled to the estimated next step duration. Hence, a shift in the error vector can lead to a different shift in the applied control signal. Therefore, the control signal $\mathbf {u}_{k+1}^{\dag }$ has to be shifted after the resampling is applied:

9 $$\begin{array}{*{20}l} \hat{\mathbf{u}}_{k+1}^{\dag} = \left[\begin{array}{c} \hat u_{k+1, m}^{\dag} \\ \vdots \\ \hat u_{k+1, \hat {N}_{\text{step}}}^{\dag} \end{array}\right],  \end{array} $$

where $\hat {\mathbf {u}}_{k+1}^{\dag }$ is the shifted control signal. When applying the control input during the next step, it can happen that the step continues for more than $\hat {N}_{\text {step}} - m$ samples. After $\hat {N}_{\text {step}} - m$ samples have passed, the control input is defined to be zero. For a constant step duration, this means losing control over the last *m* samples of the stimulation trajectory.

### Control signal decay

With these new extensions that we have just described, the ILC is able to produce a control signal that pushes the system inside of the defined reference bands. However, when the system stays inside of the reference bands using a nonzero input, it is impossible to tell if the system would also be able to stay within the bands using a smaller control signal. When applying the ILC to FES there are many reasons to use only as little stimulation as is needed. To solve this problem, an iterative way is chosen: for all points in the control signal where the error is zero at the same point, the control signal is lowered by a certain amount toward zero. Thus, the control signal always decays toward zero on points where the error is zero.

To achieve this, first the error signal is transformed to the GCP domain:

10 $$\begin{array}{*{20}l} \mathbf{e}_{k}^{*} = \underset{N_{\text{GCP}}}{\text{resamp}} (\mathbf{e}_{k}). \end{array} $$

A control signal decay vector $\Delta \mathbf {d}_{k}^{*} = [\Delta d_{k,1}^{*} \ldots \Delta d_{k,N_{\text {GCP}}}^{*}]^{T}$ is defined as

$$\begin{array}{*{20}l} &\Delta d_{k,i}^{*} = \\ &\quad\ \left\lbrace \begin{array}{ll} - \min (|u_{k,i}^{*}|, d) & \ \text{if}\ u_{k,i}^{*} > 0 \land e_{k,i}^{*} = 0 \\ + \min (|u_{k,i}^{*}|, d) & \ \text{if}\ u_{k,i}^{*} < 0 \land e_{k,i}^{*} = 0 \\ 0 & \ \text{otherwise} \end{array} \right. \\ &\forall i \in [1 \ldots N_{\text{GCP}}], \end{array} $$

where *d* is the amount of decay towards zero with each cycle. When $u_{k,i}^{*}$ is closer to zero than *d*, it is set to zero.

The decay signal is not necessarily smooth, hence it also has to be Q-filtered to guarantee ILC robustness:

11 $$\begin{array}{*{20}l} \Delta \hat{\mathbf{d}}_{k}^{*} = \mathbf{Q}^{*} \Delta \mathbf{d}_{k}^{*}, \end{array} $$

where **Q**^∗^ is a second Q-filter matrix, matching to the size of the signals in the GCP domain and $\Delta \hat {\mathbf {d}}_{k}^{*}$ is the filtered decay signal.

The learning rule (7) now has to be changed to

12 $$\begin{array}{*{20}l} \mathbf{u}_{k+1}^{*} = \overset{u_{\text{max}}}{\underset{u_{\text{min}}}{\text{sat}}} \left(\mathbf{u}_{k}^{*} + \Delta \mathbf{u}_{k}^{*} + \Delta \hat{\mathbf{d}}_{k}^{*} \right). \end{array} $$

### ILC framework

**Fig. 9:**
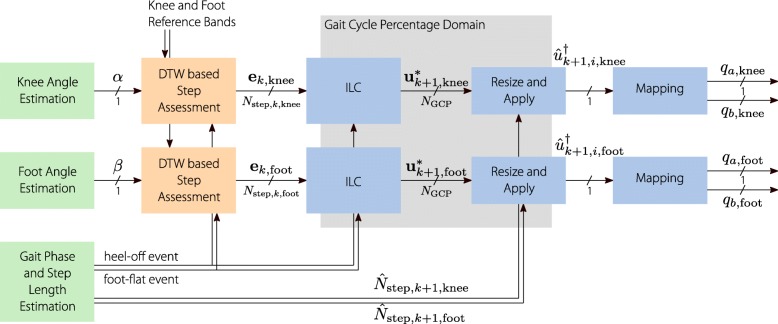
Schematic of the ILC. The knee assessment and ILC are triggered by the heel-off event. The error of the last knee angle cycle gets passed to the ILC, which generates the new control input. The control input is in the gait cycle percentage domain and has to be resized to the estimate of the duration of the next cycle. The resized control input is played back sample by sample and transformed to stimulation intensities for the antagonistic knee muscles. Equally the foot assessment and ILC are triggered by the foot-flat event

The resulting ILC framework is depicted in Fig. 9. For the knee angle and foot angle control of one leg, two independent copies of the established ILC are used. The knee angle ILC and foot angle ILC are both triggered by their respective gait events (heel-off and foot-flat). When triggered they each supply the control input for the next gait cycle in the gait cycle percentage domain. Together with the respective trigger event, this control signal is then resized to the current estimate of the cycle duration and played back, sample by sample, in real time. The two real-time control signals are mapped by the respective mapping strategies into stimulation intensities for the antagonistic muscle pairs. Here, *q*_*a*,knee_ is the stimulation intensity for the quadriceps muscle, *q*_*b*,knee_ the hamstring muscle, *q*_*a*,foot_ the tibialis anterior muscle and *q*_*b*,foot_ the gastrocnemius muscle.

## Experimental setup

The proposed neuroprosthesis was implemented, parametrized and tested with four people with an ambulatory incomplete SCI. For each participant, an automatic parameter identification procedure was conducted while sitting. Subsequently, each participant was asked to walk on a treadmill while wearing the neuroprosthesis. During this time, the prosthesis was switched on and off in one minute intervals.

### Hardware and software implementation

The hardware used in the experiment was a four-channel stimulator (Rehamove 3, Hasomed GmbH, Germany), three 9-DOF Bluetooth IMUs (RehaGait, Hasomed GmbH, Germany) and a standard PC.

Due to the wireless bandwidth limits of the Bluetooth IMU sensors, the setup could only assess and stimulate one leg. Using wired IMU sensors or a different wireless implementation would enable a symmetric two leg version of the neuroprosthesis. In the experiments, the more affected side of each participant was chosen for stimulation.

The gait phase detection, joint angle estimation, step assessment and ILC were implemented in Matlab/Simulink (partly using C/C++). The Simulink diagram was converted to C/C++ code using the Simulink Embedded Coder and run in a soft Linux real-time environment on a PC. The IMU data was sent from the sensors via Bluetooth with a frequency of 100Hz. The joint angle estimation and gait phase detection were run at the same frequency of 100Hz; the ILC and step-assessment was run with the stimulation frequency of 50Hz. The Stimulator received and executed stimulation commands via USB at a constant frequency of 50 Hz. A biphasic pulse form was chosen in which the two pulses had the current amplitude *I* and −*I* respectively and each of the pulses the pulse width *p*_*w*_. Frequencies of 20–30Hz are often seen as an optimum for minimizing fatigue [47]. When working with people with an incomplete SCI or a stroke, the maximum achievable force is mainly limited by the person’s comfort limits. Choosing higher stimulation frequencies increases the produced force with the same pulse setting [48]. Due to the potentially low comfort limits, the ability to generate sufficient force was favored above having a good fatigue to force trade-off. Hence, the stimulation frequency was set to a relatively high value of 50Hz.

At the end of each cycle, the new stimulation patterns for the next cycle should be instantly calculated. This means that the DTW calculations, together with the ILC update, can take a maximum of one sampling instance $\frac {1}{50\,\text {Hz}}=0.02\,$s. Due to the high computational complexity of DTW (approx. *O*(*N*^2^), see [45]), this goal could not be achieved. A compromise was found in which the ILC and DTW calculations were done in two sampling steps (0.04s) and the stimulation was zero for the first sample of each cycle.

### Participants

Four people with a SCI were asked to participate in a first test of the neuroprosthesis. The participants were recruited at the Hospital Nacional de Paraplejicos Toledo, Spain. The inclusion criteria were: incomplete SCI; at least three months of clinical treatment and stable clinical condition; age between 18 and 70 years; tolerance to standing; walking ability with walker and/or crutches without assistance for at least 10 minutes, at a minimum speed of 1km/h; spasticity in plantar/dorsal ankle flexors and knee flexors/extensors less than or equal to two of the Modified Ashworth Scale; and ability to follow instructions.

The exclusion criteria were: peripheral neuropathy that interferes with the effect of electrical stimulation or contraindication; metal implant or implanted medical electrical equipment; antecedents of previous surgeries in the last six months; comorbidities that affect walking and the application of electrical stimulation; history of frequent falls; debilitating disease; alteration of mental functions that prevent the subject from following instructions; and refusal to sign informed consent.

All the subjects were informed about the study and a written consent was obtained before the session. The experimental study has been carried out after the formal approval of the local ethical committee of the hospital, Hospital Nacional de Parapléjicos-Toledo, Spain (C.E.I.C – 368).

### Experimental procedure

The positioning of the stimulation electrodes can be seen in Fig. 1. The following passive gel electrodes (Axelgaard ValuTrode) were chosen for stimulation: two 5 *x* 9 cm electrodes for the quadriceps, two 5 *x* 9 cm electrodes for the hamstrings, two oval 4 *x* 6.4 cm electrodes for the tibialis anterior and two 4 *x* 9 cm electrodes for the gastrocnemius. The IMUs were attached using straps and an elastic bandage for the foot mounted IMU.

Before starting the walking experiment, an automatic procedure was conducted to identify the ILC parameters as well as the maximum painless stimulation intensities for each muscle of the individual participant. First, the participant was asked to sit on a high surface so that the concerned leg was able to swing freely. The stimulation intensity was then slowly ramped up for each channel until terminated by verbal indication of the participant. This was repeated three times for each participant. During the procedure the foot and knee angles were recorded together with the stimulation intensity. From this data, a static gain *K* [ ^*circ*^/µAs], a stimulation threshold *q*_0_ [µAs] and the maximum stimulation *q*_max_ [µAs] for each muscle was determined. This could be achieved by fitting a piecewise linear curve (constant until the threshold, then a linear gain) to the stimulation intensity/joint angle data. In the case that the participant showed very little reaction to the stimulation, *q*_0_ was limited to a maximum of 5.00µAs. For higher values of *q*_0_, the constant cocontraction stimulation can feel uncomfortable. The estimated static system gain *K* was limited to a minimum of 0.12^*circ*^/µAs. Since *K* is inverted in the static system inverse (2), values closer to zero can lead to unreasonable high and rapidly-changing stimulation intensities. This limiting of *q*_0_ and *K* was carried out after the parameters were identified.

**Fig. 10:**
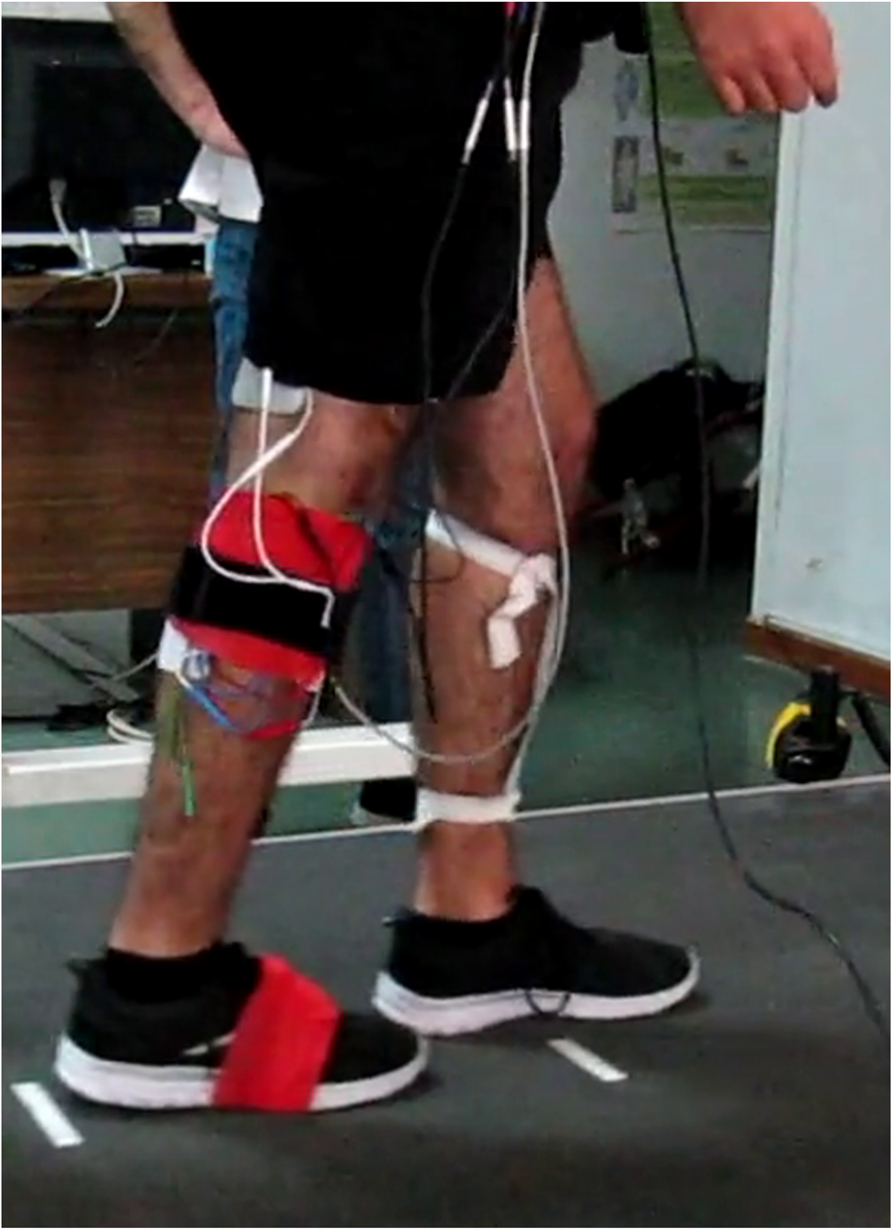
Picture of one of the people with a SCI during the experiment. The participant is walking on a treadmill. On the right leg, the stimulation electrodes and IMU sensors are partly visible. A detailed illustration of the electrode and sensor placement is given in Fig. 1

The neuroprosthesis experiment was conducted in the following way. When conducting the experiment we always chose the foot-flat based version of the foot angle control first. In the case of timing problems, the experiment was restarted with the heel-off based version. The participant was asked to stand upright on the treadmill, this instance was used to define a knee angle of 0^*circ*^. To ensure the safety of the participant, all participants were secured by a harness as well as accompanied by a therapist. First, the speed was slowly increased while consulting with the participant, until a comfortable, self selected, pace was found. The participant walked then for one minute without any stimulation. Then, the neuroprosthesis was activated and the stimulation patterns were adapted and applied (changing with every gait cycle) for another minute. This two minute procedure was repeated until the participant was tired or the therapist declared the end of the rehabilitation session. After every two minute repetition, the ILC was reset and started anew with stimulation patterns of zero intensity. A photograph of the actual measurement can be seen in Fig. 10.

### Parameters

For both, the knee and the foot ILC, the same set of fixed parameters were chosen (see Table 1). Having a person-independent set of ILC parameters was possible by setting the parameters for the input mapping so that the mapping resembles the static system inverse. The ILC can then assume a system with a static system gain of 1. The input mapping parameters *k*_*a*_, *k*_*b*_, *q*_*a*0_ and *q*_*b*0_ were set to the identified parameters of the preliminary ramp identification experiment. With this parameterization, and due to the system inverse, the control signal $\hat {\mathbf {u}}_{k}^{\dag }$ has the same unit as the measurement signal, namely degree, unlike the actual stimulation intensity signals *q*_*a*_ and *q*_*b*_, which are given in µAs.

**Table 1:** For all experiments, the ILC was tuned with the following parameters

ILC Parameters		
Plant delay	*m*	10
Max samples	*N*_step, max_	300
GCP domain samples	*N*_GCP_	200
ILC gain	*λ*	1
Decay factor	*d*	$\frac {1}{15}$
Error limit	*e*_max_	410^*circ*^
ILC Qfilter	**Q**: 1st order backward-forward butterworth, *f*_cuttoff_=5Hz	
ILC decay Qfilter	Q*: 1st order backward-forward butterworth, *f*_cuttoff_=5Hz	

The parameters for the individual participants were contained in the input mapping and set by the automatic parameter tuning procedure

We assumed a delay between the stimulation and joint angle response of 0.2s (see “Background” section and [25–27]). With the sampling frequency of 50Hz, this lead to a plant delay of *m*= 10 samples.

The ILC was limited to allow a maximum of samples per cycle *N*_step, max_. With the chosen setup the maximum cycle duration is 6s. The decay factor was chosen so that when the joint angle stays within the reference bands and the stimulation is at maximum intensity, a complete decay to zero intensity requires 15 cycles.

The ILC Q-filter matrix was created by composing a lifted system filter matrix **F** of the first *N*_step, max_ impulse responses of the filter (see [29] for details). To achieve an acausal backward-forward filtering, the Q-filter matrix **Q** was chosen to be **FF**^*T*^.

Since the ILC decay Q-filter **Q**^∗^ filters signals in the GCP domain, as opposed to the time domain, there is no meaningful unit for the sampling time. We chose to assume an average step duration of 1 second, as a consequence the sampling time is chosen 0.01 s for an *N*_GCP_ of 200.

## Results

For all four participants, the parameter identification was conducted while sitting, before starting the walking experiment. This procedure took an average of 139 s. For the first three participants, the joint angles changed significantly when ramping up the stimulation intensity, channel after channel. These three participants showed discomfort only at high levels of stimulation or no discomfort at all. For participant 3, an unusually high level of hamstring stimulation (10.49µAs, note the difference from Table 2 in which the parameter *q*_0_ was limited to 5.00µAs) was necessary to induce notable motion. Participant 4 experienced an increased pain sensation and therefore discomfort was felt at low levels of stimulation intensity (see Table 2). As a result, no visible motion could be induced except when stimulating the quadriceps. Table 2 shows the identified parameters from the automatic parameter identification for each participant. The identified system gains were set to a minimum of 0.12^*circ*^/µAs, to remain within a reasonable range. Participant 4 could only surpass this minimum with the quadriceps muscle.

**Table 2:** The automatically identified parameters

	*q*_*a*0_	*K*_*a*_	*q*_*a*max_	*q*_*b*0_	*K*_*b*_	*q*_*b*max_
	[µAs]	[ ^*circ*^/µAs]	[µAs]	[µAs]	[ ^*circ*^/µAs]	[ µAs]
	Quadriceps			Hamstrings		
Participant 1	5.00	1.55	12.20	5.00	0.28	10.03
Participant 2	3.28	2.36	8.57	2.10	0.16	14.36
Participant 3	5.00	1.92	18.00	5.00	0.49	20.99
Participant 4	5.00	1.26	9.11	3.84	0.12	10.81
	Tibialis Anterior			Gastrocnemius		
Participant 1	3.25	1.66	8.40	4.23	1.43	7.14
Participant 2	2.24	3.42	11.38	3.05	1.45	12.15
Participant 3	3.70	1.49	13.05	3.89	0.32	15.18
Participant 4	4.29	0.12	6.52	4.23	0.12	5.68

*q*_*a*0_ and *q*_*b*0_ are the stimulation intensity thresholds of one antagonistic muscle pair, *K*_*a*_ and *K*_*b*_ the identified steady state gains and *q*_*a*max_ and *q*_*b*max_ the maximum comfortable stimulation intensities for the individual muscle pair and participant. In order to translate the given charge (µAs) to the applied current and pulse width, please refer to Eq. (3)

**Fig. 11:**
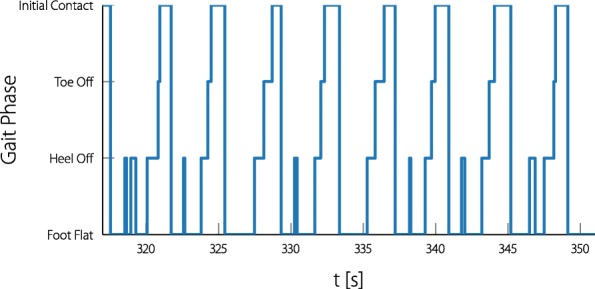
Gait phase detection issues with participant 1. Due to the many false positive heel-off detections the ILC was triggered at the wrong times and the experiment had to be aborted

Participant 1 was the most severely affected out of the four. His weight had to be supported by a harness, and a therapist walking together with the participant helped stabilizing the torso. Due to the insecurity and shaking of the leg of the participant during the stance phase, the heel-off event was triggered multiple times during each stance phase. This led to triggering of the ILC at the wrong time, leading to disruption of the gait by the resulting uncomfortable stimulation patterns. The output of the gait phase detection during this measurement is shown in Fig. 11, in which the back and forth triggering between heel-off and foot-flat can be observed. The experiment was canceled due to the inability of the gait phase detection.

With participant 2, when using the foot-flat triggered ILC for the foot angle, the participant confirmed that there were problems with the timings and the stimulation did not feel supportive. The foot ILC had to be switched to the heel-off triggered version (as described in Fig. 8), and therefore a push-off support was not possible. For participant 3 and 4 the foot-flat triggered foot ILC was used. Participants 2, 3 and 4 confirmed that the stimulation was coming at the right times and felt supportive. When activating the knee ILC for participant 2, unreasonable stimulation patterns occurred during the stance phase. Because of this issue, for participant 2, the knee angle reference band was widened during the stance phase as can be observed in Fig. 13 (compared with the original reference presented in Fig. 2). For participant 4 the stimulation limits had to be lowered further during the experiment due to discomfort.

**Fig. 12:**
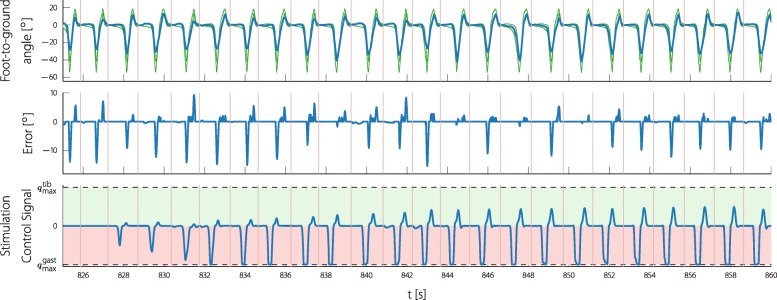
Continuous time experiment data of the foot ILC. The foot-to-ground angle and the stimulation signal are shown as seen during the experiment. The reference bands and the error signal are plotted in an acausal way, since the assessment for each cycle is done at the end of the cycle. The shown data is part of the experiment with participant 3. Note that the positive plane of the stimulation control signal was normalized to the maximum tibialis anterior stimulation intensity $q_{\text {max}}^{\text {tib}}$ (13.05µAs) and the negative plane to the maximum gastrocnemius stimulation intensity $q_{\text {max}}^{\text {gast}}$ (15.18µAs). Due to the cocontraction mapping strategy, a stimulation control signal of zero still leads to a certain stimulation. The vertical lines () mark the foot-flat events

**Fig. 13:**
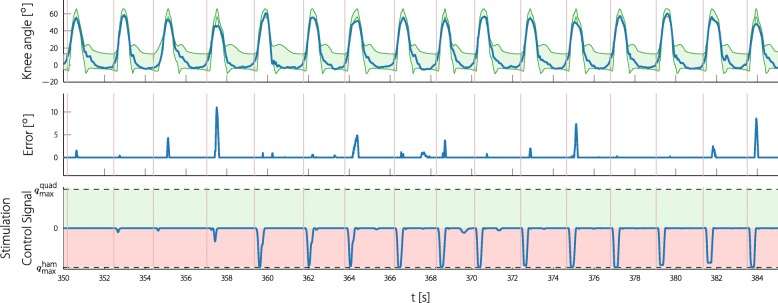
Continuous time experiment data of the knee ILC. The knee angle and the stimulation signal are shown as seen during the experiment. The reference bands and the error signal are plotted in an acausal way, since the assessment for each cycle is done at the end of the cycle. The shown data is part of the experiment with participant 2. Note that the positive plane of the stimulation control signal was normalized to the maximum quadriceps stimulation intensity $q_{\text {max}}^{\text {quad}}$ (8.57µAs) and the negative plane to the maximum hamstrings stimulation intensity $q_{\text {max}}^{\text {ham}}$ (14.36µAs). Due to the cocontraction mapping strategy, a stimulation control signal of zero still leads to a certain stimulation. The vertical lines () mark the heel-off events

In Figs. 12 and 13 one example of the knee ILC and one of the foot ILC is shown during the measurement. The shown recording of the foot ILC starts shortly before the ILC is switched on, so that the learning process can be observed. The upper row shows the measured foot angle and the generated reference bands; the second row shows the foot error produced by the automatic gait assessment. Note that the reference and the error signal is shown in an acausal way, since the automatic gait assessment produces the entire reference and error vector after each step. The stimulation input (seen on the bottom rows) is shown as applied to the participant by the ILC during the experiment. When looking at the error, it can be noted that in almost every step, the participant lacks push-off during the pre-swing phase as well as dorsiflexion during the terminal swing phase. The applied stimulation control signal converges step by step to a fixed pattern.

A similar example is shown of the knee ILC (Fig. 13) where the stimulation control signal also converges. This time the assessment suggests too little knee flexion during swing and the ILC is stimulating the hamstring muscles during swing with the maximum tolerated stimulation.

**Fig. 14:**
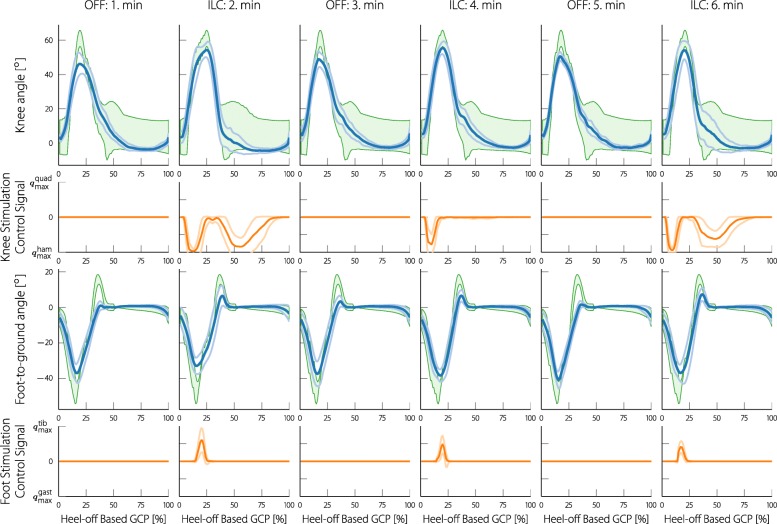
Mean and standard deviations of the knee and foot joint angles of participant 2 during the first six minutes of the experiment. For the first minute the neuroprosthesis was turned off, for the second minute it was turned on, and so forth. The joint angles were all resampled to the gait cycle percentage domain. The green areas in the background are the reference bands that were used during the experiment (for participant 2 a wider knee reference band was used). The reference bands are fitted to the mean joint angles using DTW similarly to how the reference is fitted to each individual step in the real-time gait assessment. In this representation, the gait cycle is started with the heel-off event for both the knee and the foot angle. Below the joint angles, the respective stimulation control signals (mean and standard deviation) are presented. For the knee a positive control signal implies quadriceps stimulation and a negative signal hamstring stimulation. For the foot control signal, positive values imply tibialis stimulation and negative values gastrocnemius stimulation. The positive and negative planes of the control signal were scaled to the maximum tolerated stimulation for the respective muscle of the participant (the values can be found in Table 2)

**Fig. 15:**
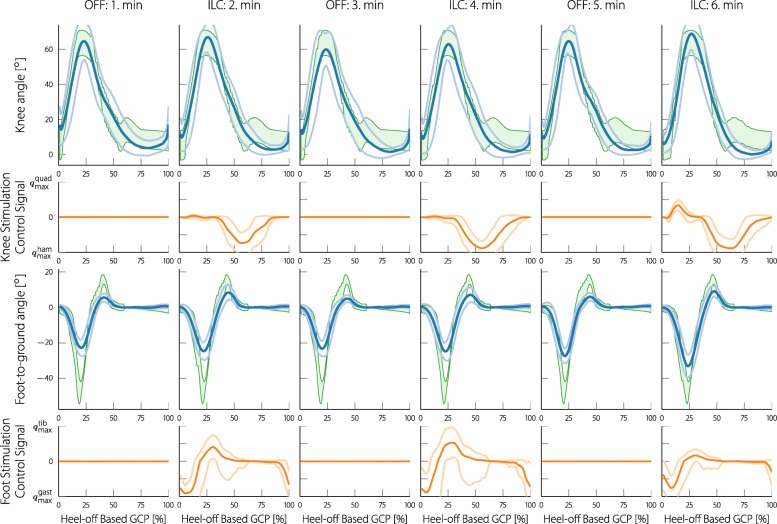
Mean and standard deviations of the knee and foot joint angles and stimulation control signals of participant 3 during the first six minutes of the experiment. In this representation, the gait cycle is started with the heel-off event for both the knee and the foot angle

As experiments were alternated with one minute of no FES and one minute of activating the neuroprosthesis, for each of the minute intervals, the joint angles were averaged and shown together with their standard deviations. Figure 14 shows the result for participant 2 and Fig. 15 for participant 3. Along with the mean and standard deviations, reference bands are shown that were used in the respective experiments. These reference bands were warped (by using the same method as in the assessment) to match the presented mean joint angles. With participant 2, the foot dorsiflexion during the terminal swing phase, as well as the knee flexion during the swing phase is visibly increased when the stimulation is turned on. For participant 3, both foot dorsiflexion during terminal swing and push-off are increased, whereas no improvements can be seen in the knee angle. In addition to the joint angles, the mean and standard deviations of the stimulation control signal are shown below the respective joint angle. The presented control signal was normalized to the maximum allowed stimulation intensities. The stimulation patterns indicate hamstring stimulation during knee flexion for participant 2, and hamstring stimulation during the stance phase of participant 2 and 3. The push-off of participant 3 is supported by gastrocnemius stimulation and the terminal swing of participant 2 and 3 are supported by tibialis stimulation. Due to the low levels of stimulation and the lacking change of gait, a figure for participant 4 is not presented.

These results are also numerically presented in Table 3. The mean Root Mean Square (RMS) error, as well as the mean minimum and maximum error, are shown separately for the times with and without stimulation. Additionally, the ratio of the RMS error with and without stimulation is shown. As the RMS error is calculated over the entire step circle, low values are to be expected since the errors arise only during short periods (for example, push-off and terminal swing). With the knee angle, a high *E*_max_ means a lacking knee flexion and a high negative *E*_min_ a lacking knee extension. With the foot-to-ground angle, a high *E*_max_ means a lacking dorsiflexion and a high *E*_min_ a lacking push-off (plantar flexion). Thus, for participant 2, the foot dorsiflexion was increased by an average maximum of approximately 4^*circ*^, and the plantarflexion was increased by a average maximum of approximately 2^*circ*^. For participant 3 these average maximum improvements were approximately 3^*circ*^ and 4^*circ*^, respectively. The knee flexion of participant 2 was increased by an average maximum of approximately 4^*circ*^. No further significant improvements could be measured. Furthermore, the self selected treadmill walking speeds of the participants, as well as the passed gait cycles are presented in the table.

**Table 3:** Mean values of the RMS error of each step (*E*_RMS_) taken with and without stimulation

		Participant 2		Participant 3		Participant 4	
Minutes		5	5	7	7	7	7
Status		Off	On	Off	On	Off	On
Knee	*E*_RMS_ [ ^*circ*^]	1.58	0.77	2.41	2.42	2.48	2.49
	*R*_on/off_ [%]		48 %		100 %		100 %
	*E*_max_ [ ^*circ*^]	7.85	3.46	4.83	4.89	3.67	5.38
	*E*_min_ [ ^*circ*^]	-0.15	-0.27	-5.02	-5.2	-7.30	-6.35
Foot	*E*_RMS_ [ ^*circ*^]	3.78	2.93	3.15	2.03	6.99	6.50
	*R*_on/off_ [%]		78 %		65 %		93 %
	*E*_max_ [ ^*circ*^]	15.03	10.79	5.33	3.47	21.99	20.30
	*E*_min_ [ ^*circ*^]	-2.76	-5.77	-13.23	-8.60	-4.08	-3.92
Cycles		288		625		403	
Speed [km/h]		1.3		1.9		1.0	

*R*_on/off_ is the ratio of (*E*_RMS_) with stimulation to without stimulation of the corresponding participant. Mean maximum error values of each step (*E*_max_) taken with and without stimulation. Mean minimum error values of each step (*E*_min_) taken with and without stimulation. The cycles are the total gait cycles passed during the experiment and the speed is the chosen walking speed on the treadmill by the participant

## Discussion

In a first test, four people with a SCI were asked to walk with the proposed neuroprosthesis. For three of the participants, the stepwise-generated stimulation patterns felt supportive and well timed. For two participants, slight changes towards the desired reference bands could be measured; one participant was more severely impaired which led to a false positive detection of heel-off events and one participant could not be functionally stimulated due to high pain sensation.

The automatic parameter identification includes the essential setting of the participant’s comfort limits and prevents any manual setting of parameters. With an average duration of 139 s, it can be realistically included into a rehabilitation setting.

The measurement with participant 1 was quickly aborted due to the false positives of the gait phase detection. The gait phase detection from [39] can be tuned by many parameters and the problem could have been likely solved by raising the threshold (*α*_PS_) for the heel-off detection. However, manual tuning of the gait phase detection is not an aim for a practical setting of the neuroprosthesis. As with the other three participants, the gait phase detection worked as expected, as the four gait phases were passed consecutively in the correct order throughout the experiment.

The proposed gait assessment was able to adapt the reference joint angle bands to the individual gait of the participant. The reference bands naturally follow the foot and knee angle of the participants (as shown in Figs. 12 and 13), and therefore meaningful joint angle errors can be provided. Matching the reference to the joint angles of the participant is an organic process that makes a quantitative evaluation difficult. The errors reflecting the typical drop foot problem during swing phase and the lacking push-off during pre-swing, as well as the resulting logical stimulation patterns, indicate a success of the proposed gait evaluation method.

The knee and the foot ILC converge to a repeating non-trivial stimulation pattern. When looking closely at the pattern of the foot (Fig. 12) it can be observed that it reaches the maximum gastrocnemius stimulation in the pre-swing phase and toward approximately a third of the maximum stimulation of the tibialis anterior muscle during swing phase. This closely resembles the natural activation of these muscle groups during gait (see for example, [49]).

The new stimulation control pattern, which is generated for every step, is shorter than the expected step duration due to a time shift to compensate the slow FES dynamics, see (9). Hence, for the last samples of most steps, the stimulation control signal is set to zero. A sudden change of stimulation intensity could disrupt the current motion or could feel unpleasant. Due to the choice of gait events for the triggering of the ILC (foot-flat or heel-off), we expected little or no control action during this time. As can be observed in Figs. 12 and 13 there was no issue with sudden drops of the stimulation intensity at the end of an ILC cycle.

As we have already described, for the knee angle reset the participants were asked to stand straight and the angle was defined to be zero in this position. The knee angle assessment turned out to be very sensitive to this reset. If the participant slightly hyperextended or slightly flexed the knee during reset, it was hard for the therapist to notice. This change of a few degrees often meant that during loading response and mid-stance, the knee angle was slightly above or below the reference band, leading to increased stimulation in this phase. When examining the recorded joint angle it was hard to see if the angle was wrongly calibrated or if the gait of the participant deviated from the norm. As we have already mentioned, the knee reference band had to be widened during the stance phase for participant 2 (see Fig. 13) to account for this problem. Widening the reference bands, however, reduces the FES support during stance phase (a wider reference leads to a smaller or no error). Consequently, optimal knee FES support during stance phase is not reliably possible with the current solution. In Figs. 14 and 15 it is evident that often, the knee angle is below the reference during stance phase, indicating knee hyperextension. This lead to a stimulation of the hamstrings during the stance phase by the ILC. While this stimulation pattern might seem counterintuitive for weight acceptance, Springer et al. could show that FES of the hamstrings is beneficial for people with knee hyperextension [50].

When looking at the knee stimulation pattern in Fig. 13, a problem with the ILC gain can be seen. The stimulation pattern jumps from almost no stimulation in one step to the maximum amount of stimulation in the next. As shown in the method section, the error of the ILC is limited, which means that the amount of input change from step to step is also limited. The aim of this neuroprosthesis is to learn a stimulation pattern and to not react extremely to a single odd step. This means that the ILC gain in this scenario was chosen too high by the automatic system identification. As we have explained before, the ILC gain is chosen for each muscle individually by estimating the static system gain of each muscle. This resulted in a parametrization of the ILC, which proved not to lead to the desired ILC learning rate in many cases. A better method might be to tune the ILC so that with the maximum allowed error, the maximum allowed stimulation is reached after a set number of steps (for example, five). This would also further simplify and shorten the identification procedure.

In the classic ILC applications, with every cycle the error is supposed to decrease, eventually reaching a certain minimum level. When looking at the two examples, it is evident that the error fluctuates with every step and does not necessarily decrease. Since the applied stimulation control input was repetitive and well timed, it is safe to assume that the error fluctuation emerges from the complex gait process and voluntary muscle interaction. Therefore, it should not be individually analyzed but rather statistically processed, as was done in Figs. 14 and 15 and Table 3. If the learning gain of the ILC is low enough, the statistical properties can be smoothed out and the control signal can converge as it did in the presented measurements.

In the statistical evaluations of Figs. 14 and 15 and Table 3, slight improvements of the averaged joint angles could be observed for participant 2 (knee and foot) and participant 3 (foot improvements only). These averaged maximum improvements were in the range of 4^*circ*^. For participant 4, as would be expected with the non-functional stimulation levels, the joint angles could not be improved. The increased standard deviations in the minutes with stimulation can be explained by the slow learning of the ILC. This slow learning leads to changing stimulation patterns (and therefore reactions) during the first part of the minute.

Altogether, relatively small statistical changes of the gait were achieved. However, cyclically decreasing errors or big angular improvements could not be observed. The change of, for example, the mean maximum foot error of participant 2 from 15.03^*circ*^ to 10.79^*circ*^ might not seem like a big change, but should be seen in the context of the aim and limitations of the proposed FES neuroprosthesis. Firstly, it can be seen that, if necessary, the prosthesis increases stimulation intensity up to the maximum allowed amount (see Figs. 12 and 13). If the maximum achievable support by FES is reached, if the timing is correct, any other control strategy can not push the joint angles further towards the desired gait trajectory. Secondly, rehabilitation is not a sudden change but a process. Guiding the pathological gait of a person more towards the gait of a healthy person is our main aim and can, to some extent, be achieved by this neuroprosthesis. Providing the participant with a direct biofeedback that is not only felt, but that also acts on four important muscle groups of the gait process, can be a step towards improved rehabilitation. The proposed neuroprosthesis directly reacts to any change of the gait of the participant and supplies new customized stimulation patterns with every step. This dynamic and direct feedback to the participant distinguishes this research from the prevalent simple triggered stimulation approaches.

## Conclusion

In this paper the first approach for an adaptive full-cycle full-leg support FES neuroprosthesis was presented. This neuroprosthesis can be seen as a next step to the previously published FES solutions. Learning of stimulation patterns was already achieved in [26, 27, 32], in which a single muscle (tibialis anterior) or a synergetic muscle pair (tibialis anterior and peroneus longus) were controlled exclusively during the swing phase. In both cases, the reference could not adapt to the pace or way of gait of the participants. In works including the stimulation of the full leg [11–13, 16, 18, 19], the stimulation patterns were fixed (in shape and intensity) and could not adapt to any changes in the gait of the participants (aside from adaptions to step duration).

A first test was conducted with four people with ambulatory incomplete SCI walking on a treadmill. The measured data showed that the neuroprosthesis could assess the joint angles and generate suitable individual stimulation patterns for the four targeted muscle groups of the participants. Two participants reported that they felt supported by the stimulation at the right times. For those participants, slight improvements of the averaged joint angles could be observed. A steady gait and a minimum level of muscle activation by the FES proved to be essential for an effective neuroprosthesis; these factors were not present with the two participants who did not feel supported by the neuroprosthesis. Remaining problems include the sensitivity to the knee angle reset, timing problems in participants with significant gait fluctuations, and the automatic ILC gain tuning.

Future studies should investigate a two-sided implementation of the neuroprosthesis on a higher number of people with a SCI, and a one-sided implementation for people with a stroke. In the tests presented here, only one measurement was conducted per participant. However, in future, measuring over a longer period of time and comparing with a control group could show more significant gait improvement.

A novel method of gait assessment has been proposed in this paper that allows an immediate continuous joint angle assessment for each step of the participant. This method could be applied to achieve automated clinical gait assessment, biofeedback, or gamification of rehabilitation training. Future work could investigate recording different sets of reference joint angles with a bigger number of subjects, for different age groups, and for a wider range of walking speeds.

## Data Availability

The datasets used and/or analysed during the current study are available from the corresponding author on reasonable request.

## Abbreviations

DTW: Dynamic time warping
EMG: Electromyography
FES: Functional electrical stimulation
GCP: Gait cycle percentage
ILC: Iterative learning control
IMU: Inertial measurement unit
R2R: Run to run control
SCI: Spinal cord injury
SISO: Single input single output
